# Neural underpinnings of processing combinatorial unstated meaning and the influence of individual cognitive style

**DOI:** 10.1093/cercor/bhad261

**Published:** 2023-08-09

**Authors:** Yao-Ying Lai, Hiromu Sakai, Michiru Makuuchi

**Affiliations:** Graduate Institute of Linguistics, National Chengchi University, Taipei, Taiwan; Faculty of Science and Engineering, Waseda University, Tokyo, Japan; Section of Neuropsychology, Research Institute, National Rehabilitation Center for Persons with Disabilities, Tokorozawa, Japan

**Keywords:** semantic composition, individual autistic tendency, combinatorial unstated meaning, dual-process accounts, language processing

## Abstract

We investigated the neurocognitive mechanisms underlying the processing of combinatorial unstated meaning. Sentences like “*Charles jumped for 5 minutes.*” engender an iterative meaning that is not explicitly stated but enriched by comprehenders beyond simple composition. Comprehending unstated meaning involves *meaning contextualization*—integrative meaning search in sentential-discourse context. Meanwhile, people differ in how they process information with varying context sensitivity. We hypothesized that unstated meaning processing would vary with individual socio-cognitive propensity indexed by the Autism-Spectrum Quotient (AQ), accompanied by differential cortical engagements. Using functional magnetic resonance imaging, we examined the processing of sentences with unstated iterative meaning in typically-developed individuals and found an engagement of the fronto-parietal network, including the left pars triangularis (L.PT), right intraparietal (R.IPS), and parieto-occipital sulcus (R.POS). We suggest that the L.PT subserves a contextual meaning search, while the R.IPS/POS supports enriching unstated iteration in consideration of event durations and interval lengths. Moreover, the activation level of these regions negatively correlated with AQ. Higher AQ ties to lower L.PT activation, likely reflecting weaker context sensitivity, along with lower IPS activation, likely reflecting weaker computation of events’ numerical-temporal specifications. These suggest that the L.PT and R.IPS/POS support the processing of combinatorial unstated meaning, with the activation level modulated by individual cognitive styles.

## Introduction

### The processing of unstated sentential meaning

The meaning of a sentence is largely conveyed by explicit linguistic cues, such as words, morphological markers, and syntactic structures that inform the relations among phrases at the compositional level. This is not always the case, as the full sentence meaning can go beyond straightforward composition by morphosyntactic means. This study targets such “unstated/implicit” sentential meaning—meaning that is not morpho-syntactically stated yet well understood, investigating how people process it and how the comprehension is implemented in the brain.

The investigation of unstated meaning is of special importance in advancing our understanding of language comprehension. At the sentential level, it is often difficult to separate semantics from syntax, since changes of syntactic structure usually co-vary with changes in the semantic representation. Therefore, while the neural underpinnings of sentence processing have been extensively studied, whether the observed brain activity reflects syntactic structure building or semantic composition is subject to debate. To disentangle the neural mechanisms for semantic composition from those for syntactic structure building, we target a case of unstated combinatorial meaning like (1) where the full sentence meaning goes *beyond* simple semantic composition, in which the sentence meaning results transparently from the meaning of lexical items and the way they are syntactically combined (Partee 1995).

(1) *The athlete jumped for 20 minutes at the gym.*

Example (1) gives rise to an iterative reading with multiple jumping events, in spite of the fact that the iterative meaning is not explicitly stated by any word, nor does it result from a particular syntactic structure—termed “unstated iteration” hereafter. In comparison, (2) below has an identical syntactic structure yet engenders a continuous reading with a one-time swimming event. Thus, sentences with unstated iterative meaning provide an opportunity to examine the neural correlates subserving selectively and exclusively for meaning composition independent of morphosyntax.

(2) *The athlete swam for 20 minuites at the gym.*

Previous studies have shown that sentences involving unstated iterative meaning like (1) engender additional processing cost than their transparent counterparts like (2), being found cross-linguistically in English (e.g. Piñango et al. 1999; Todorova et al. 2000) and Japanese (Long et al. 2010; Lai et al. 2019). Nonetheless, the underlying mechanisms are at debate and the neurological findings are divergent. Researchers have reported that processing unstated iteration recruited Wernicke’s area (Piñango and Zurif 2001: lesion study with aphasic patients), the ventral medial prefrontal cortex (Brennan and Pylkkänen 2008: magnetoencephalography (MEG), or the frontal-temporal/parietal regions (Piñango et al. 2016: functional magnetic resonance imaging (fMRI)). In Downey’s (2006) electroencephalography (EEG) study, sentences like (1) elicited a long-lasting negativity starting from 300 to 1,000 ms after the *for*-adverbial was encountered. Paczynski et al. (2014) also found a sustained negativity starting from 500 ms post-verb onset in sentences like “*For several minutes the cat pounced on the rubber mouse.*” without an N400 effect. Similarly, Long’s (2010) study in Japanese showed a negativity response 350–750 ms after the verb onset (appearing post-adverbially in Japanese) for compositions involving an anomaly in the temporal representation, with a frontal-focused distribution distinct from the typical centrally distributed N400 effect.

The conventional approach captures the additional cost and neural activity in (1) by assuming a mismatch in the [punctual verb + durative adverbial] composition; the mismatch is resolved by a semantic operation that coerces the punctual event denoted by the verb into an iterative reading (e.g. Jackendoff 1997; Piñango et al. 1999). However, this approach fails to explain the unstated iteration that emerges in the configuration without the presumed mismatch, as in (3).

(3) *The athlete* [*swam*]_durative_ [*for 2 months*]_durative_  *at the gym.*

Importantly, all sentences involving unstated iteration—with or without the postulated configurational mismatch—engendered comparable cost that was greater than the transparent counterparts (Piñango et al. 2016; Lai et al. 2019). In addition, the divergent findings may in part result from the heterogeneity of stimuli. While previous studies vary in the temporal adverbials they used (e.g. *for 20 minutes, until dawn, all day long*), Downey (2006) reported that *for*-adverbials diverged from others in event-related potential (ERP) profiles when composed with a punctual verb, suggesting an issue of heterogenous stimuli.

Taking these observations into consideration, Deo and Piñango (2011) proposed a Context-Dependence approach to capture the unstated iteration in *for*-adverbial sentences like (1) and (3), with and without a mismatch, in a unifying manner. Comparing examples (1)–(3), one can observe that the iterative meaning emerges essentially at the *combinatorial* level without hinging on a specific verb type. To obtain the unstated iteration, comprehenders have to evaluate the sentential context, including the event denoted by the verbal predicate plus the interval denoted by the temporal adverbial, and discourse if available. On this approach, the computational mechanisms should involve a search of conceptual representations of the received items and the integration of available information in the sentential and discourse context (cf. Jackendoff and Wittenberg 2014). We call this process of construing meaning via a search in the sentential-discourse context “meaning contextualization.”

### The computation of unstated meaning and individual differences

In addition to linguistic input, language comprehension also relies on comprehenders’ general cognitive capacity (e.g. working memory, cognitive control, etc.). Interpreting sentence meaning beyond explicit lexical and morphosyntactic markers is subject to substantial individual variability (Farmer et al. 2012; Christianson et al. 2017; Kidd et al. 2018; Bendtz et al. 2022). That is, people likely compute combinatorial meaning in different manners, using different strategies to achieve full comprehension. Since individual variability is most prominent when contextual evaluation is involved (e.g. Nieuwland and Van Berkum 2006, Nieuwland et al. 2010; Yang et al. 2018; Zhang et al. 2022), it is likely associated with the manner that comprehenders make use of contextual cues and integrate multiple lexical representations to enrich the unstated meaning.

Notice that Typically-Developed individuals (TDs) differ from each other in context-dependent meaning computation as a function of a social propensity index, the Autism-Spectrum Quotient (AQ, Baron-Cohen et al. 2001). The effect of AQ is observed in pragmatic processing, such as indirect speech (Bendtz et al. 2022), pragmatic implicature (Nieuwland et al. 2010; Yang et al. 2018), irony (Spotorno and Noveck 2014), and humor (Aykan and Nalçacı 2018). These cases all require sufficient context sensitivity of comprehenders to integrate multiple lexical representations and evaluate against the sentential-discourse context. Recent studies have demonstrated that individual differences in context sensitivity during combinatorial meaning comprehension significantly correlated with AQ (Zhang et al. 2022; Lai and Lai 2023). Note that AQ is a continuous variable graded among individuals, including both TDs and individuals with Autism Spectrum Disorder (ASDs; Baron-Cohen et al. 2001; Bishop 2012; Ruzich et al. 2015). TDs with higher AQ scores are closer to the clinical ASD group than lower-AQ TDs in socio-cognitive propensity. Accordingly, we hypothesize that language comprehension requiring a greater level of meaning contextualization would be more effortful to TDs with higher AQ scores, who are characterized by weaker context sensitivity, than lower-AQ TDs.

This perspective follows from a series of studies showing that individual autistic tendency varies with neural activity during language processing, between TDs and individuals with ASD in particular. Compared to TDs, ASDs exhibit weaker activity in the Broca’s area yet stronger activity in the Wernicke’s area of the left hemisphere during the processing of lexical semantics (Harris et al. 2006), sentential meaning (Just et al. 2004), and figurative expressions (reviewed in Mody and Belliveau 2013). Williams et al. (2013) report that TDs, but less so for ASDs, showed increased activation in the left pars triangularis (L.PT) and left middle frontal gyrus (L.MFG) in comprehending ironic (vs. literal) sentences. These results point to differential recruitments of the language network in ASDs and TDs during context-dependent meaning computation.

Aside from the ASD vs. TD group differences, the neural mechanisms of variability within TDs are less studied, but there are some suggestive findings. Nieuwland et al. (2010) report that TDs with lower AQ scores (on the Communication subscale specifically), but not those with higher AQ, showed an N400 effect to under-informative statements that triggered pragmatic implicature (e.g. *Some people have lungs.*). They suggest that high-AQ TDs tend to make reference to logical reasoning and thus are more likely to under-use pragmatic-contextual evaluation based on general world knowledge, showing weaker sensitivity to underinformativeness of the sentences. Following this, we predict that individual variability in AQ would elicit differential brain activation during the processing of unstated meaning in TDs (detailed later).

The Dual-Process Accounts (DPA; Evans 2003, 2008; Evans and Stanovich 2013; Tversky and Kahneman 1974; Kahneman 2002) may be able to capture the difference in the processing profiles among individuals varying in autistic tendency. The DPA argues that human cognitive systems can be characterized as a balance between the intuitive and associative route (System 1), in which decision making is automatic, rapid, and relatively effortless, and the deliberative reasoning route (System 2), which is slow, rule-governed, controlled and relatively effortful. We assume that individuals with higher autistic tendency rely more on deliberative reasoning over intuition, and less autistic individuals show the reverse pattern (de Martino et al. 2008; Evans 2008; Brosnan et al. 2014, 2016, 2017; Happé et al. 2017; Ashwin and Brosnan 2019; Lewton et al. 2019; Rozenkrantz et al. 2021). Mottron et al. (2006) and Baron-Cohen et al. (2009) indicate that autistic individuals exhibit hyper-attention or hyper-sensitivity to low-level, rule-based input, likely accompanied by diminished or optional higher-order computation. In comparison, people with lower autistic tendency tend to engage associative, heuristic mechanisms (i.e. System 1), thereby being better at contextual integration. The System 1, by taking a larger scope of information into consideration, seems advantageous for computing context-dependent unstated meaning at the compositional level.

We argue that social cognitive propensity indexed by autistic tendency (i.e. AQ) factors into online language comprehension, and that it would modulate the processing of unstated meaning with differential cortical recruitments in regions that support meaning contextualization. Also, we expect that the different patterns of language processing between ASD and TDs observed in previous studies apply to TDs with high and low AQ scores as well. This view provides a plausible explanation accounting for individual differences that have long been observed in real-time sentence processing (e.g. Kidd et al. 2018). The differential reliance on the dual process—intuitive thinking versus deliberative reasoning—leads to individual differences in sentence processing.

### The current study: Neural mechanisms of unstated meaning and individual variability in autistic traits

We aim to examine the neural mechanisms of processing unstated meaning at the combinatorial level, and investigate the brain activation patterns that underpin individual variability during this process. We take AQ as an indicator of cognitive styles following the DPA: higher-AQ individuals (i.e. more autistic-like) have a stronger tendency toward deliberative reasoning with less context-dependent consideration, while lower-AQ individuals (i.e. less autistic-like) are prone to associative intuition with greater context sensitivity. The novelty of the current study lies in two aspects. First, we investigate variability in cortical activation that corresponds to graded autistic tendency in TDs, rather than the dichotomic comparison between ASDs and TDs. Second, we probe this individual variability through the case of unstated combinatorial meaning that extends beyond typical pragmatic cases, which previous studies have been focusing on. To our knowledge, this is the first study that investigates the neural correlates underlying individual autistic tendency during sentence comprehension that pinpoints semantic composition.

We specifically probe unstated iteration in semantic composition for the following reasons. Individual variability in social skills has been found mainly in meaning computation that goes beyond morphosyntax, for which individuals with ASD are relatively intact (Tager-Flusberg et al. 2005). The impact of individual variability in socio-cognitive tendency is more prominent in pragmatic processing, shown in the ASD vs. TD group differences (e.g. Kalandadze et al. 2018) as well as within TDs (e.g. Nieuwland et al. 2010; Yang et al. 2018). The role of social tendency in the computation of compositional meaning, on the other hand, is less studied, and we aim to fill this gap. Nonetheless, it has been a challenge to dissociate compositional semantic computation from syntactic one. Studies that attempt to isolate semantic processing from syntactic processing often implement a violation-based paradigm or syntactic complexity (Friederici et al. 2003; Kuperberg et al. 2000; Newman et al. 2003; Zhu et al. 2009, see Rodd et al. 2010 for a similar point), and thus the observed brain activity could possibly reflect a domain-general anomaly detection process not specific to linguistic processing or more syntax-related computation. In this study, we scrutinize unstated meaning without invoking any syntactic confounds or violation-associated processes, singling out the neural mechanisms selectively and exclusively pertaining to combinatorial semantic processing. Noticeably, a recent behavioral study reveals that social cognitive propensity in TDs, indexed by AQ, significantly correlated with the time of processing unstated iterative meaning (Lai and Lai 2023). We aim to examine neural correlates underlying such individual variability in computing unstated meaning.

Our hypothesis is two-fold. First, the processing of unstated meaning would recruit brain regions that support the context-dependent meaning search for computing the unstated event iterativity (i.e. the number of event occurrences). According to the above-mentioned findings in literature, we predict that such meaning search would engage the left inferior frontal gyrus (LIFG). In addition, the regions that support numerosity may participate in the process of computing event iterativity as well, likely the bilateral intraparietal sulcus (IPS) (e.g. Dehaene et al. 2003; Dormal et al. 2008; Piazza and Izard 2009). Second, we hypothesize that the DPA is capable of capturing individual variability in language processing profiles. Higher-AQ individuals (indexing lower socio-cognitive propensity) would rely more on deliberative reasoning using rule-based computation, and are weaker at context integration (Lai and Lai 2023; Zhang et al. 2022; cf. the complex information processing account to autism by Minshew and Goldstein 1998). In terms of sentence comprehension, this amounts to a bias toward transparent compositionality, such that individuals with higher AQ scores tend to obtain sentence readings by the meanings of the given words and the straightforward integration according to morphosyntactic rules. Information not given by invariant local mechanical rules, like world knowledge or a larger scope of context, is less considered. This leads to a weaker tendency of interpreting context-dependent meaning that goes beyond explicitly stated information. Such tendency accounts for previous findings that high-AQ TDs and people with ASD encounter greater difficulty in pragmatic processing. In connection to the first point, this individual variability in processing unstated meaning would likely correspond to differential engagements in cortical regions that support context-dependent meaning search (LIFG) and the computation of event numerosity (IPS).

Note that previous work on sentential unstated iteration compared sentences like (1) with the counterparts involving the one-time reading like (2); no study has compared unstated vs. explicitly stated iteration like (4) in which the number of event iteration is lexically specified [Paczynski et al. (2014) implemented frequentative adverbials like “*several times*” without exact numbers for unstated iteration and did not examine explicitly stated once as in (4)].

(4) *The athlete jumped* [*once/10 times*] *at the gym.*

Although (2) yields a non-iterative reading, the one-time event occurrence is not expressed explicitly as in (4); we may call cases like (2) “*unstated once*” accordingly. In terms of determining iterativity, it is possible that these sentences still involve some more computation than sentences that explicitly state iterativity by lexical means like (4). The latter thus serves as a more direct baseline to be contrasted with sentences involving unstated iteration. Here we examine the neural network underlying the comprehension of unstated and explicitly stated combinatorial meaning, including both *iterative* and *once* sentence interpretations (see Materials).

## Methods

### Materials

We constructed 40 sets of four critical conditions with *kan*-adverbials (*
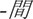
*) in Japanese (comparable to the temporal *for*-adverbials in English), crossing two verb types (punctual, durative) and two interval lengths of the temporal *kan-*adverbials (short, long) as shown in Table 1. The no-iteration (NoIter) condition consisted of a durative verb and a short-interval adverbial (e.g. *swam for an hour*), yielding an unstated *once* reading. In comparison, the unstated iteration condition with a punctual verb (PuncIter) consisted of a verb denoting a point-like action and a short-interval adverbial (e.g. *jumped for an hour*). The other two conditions involving unstated iteration contained a *kan*-adverbial that denoted a longer interval in composition with a durative verb (DurIter) and a punctual verb (GapIter). Crucially, the three iteration (ITER) conditions—PuncIter, DurIter, and GapIter—all involved unstated iterative meaning at the compositional level, regardless of verb type.

**Table 1:** Sample stimuli.

Verb Type	Interval Length	Condition	Sample sentences						
			Seg.01	Seg.02	Seg.03	Seg.04	Seg.05	Seg.06	Seg.07
Durative	Short	**NoIter**	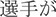	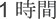	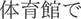	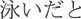	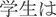	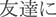	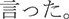
		Unstated Once	senshu-ga	ichiji-kan	taiikukan-de	oyoida-to	gakusei-wa	tomodachi-ni	itta
			athlete-NOM	1 h for	gym-LOC	swim-PAST-COMP	student-TOP	friend-DAT	say-PAST
			“The student said to his/her friend that the athlete swam for an hour at the gym.”						
Punctual	Short	**PuncIter**	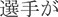	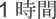	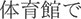	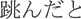	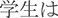	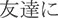	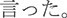
		Unstated Iteration	senshu-ga	ichiji-kan	taiikukan-de	tonda-to	gakusei-wa	tomodachi-ni	itta
			athlete-NOM	1 h for	gym-LOC	jump-PAST-COMP	student-TOP	friend- DAT	say-PAST
			“The student said to his/her friend that the athlete jumped for an hour at the gym.”						
Durative	Long	**DurIter**	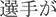	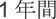	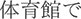	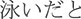	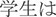	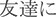	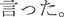
		Unstated Iteration	senshu-ga	ichinen-kan	taiikukan-de	oyoida-to	gakusei-wa	tomodachi-ni	itta
			athlete-NOM	1 year-for	gym-LOC	swim-PAST-COMP	student-TOP	friend-DAT	say-PAST
			“The student said to his/her friend that the athlete swam for a year at the gym.”						
Punctual	Long	**GapIter**	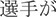	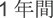	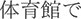	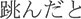	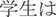	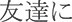	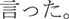
		Unstated Iteration	senshu-ga	ichinen-kan	taiikukan-de	tonda-to	gakusei-wa	tomodachi-ni	itta
			athlete-NOM	1 year-for	gym-LOC	jump-PAST-COMP	student-TOP	friend-DAT	say-PAST
			“The student said to his/her friend that the athlete jumped for a year at the gym.”						
		**Once**	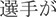	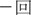	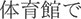	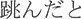	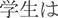	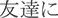	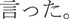
		Explicit Once	senshu-ga	ik-kai	taiikukan-de	tonda-to	gakusei-wa	tomodachi-ni	itta
			athlete-NOM	1-time	gym-LOC	jump-PAST-COMP	student-TOP	friend-DAT	say-PAST
			“The student said to his/her friend that the athlete jumped once at the gym.”						
		**Multiple**	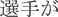	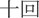	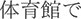	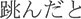	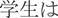	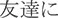	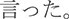
		Explicit Iteration	senshu-ga	jik-kai	taiikukan-de	tonda-to	gakusei-wa	tomodachi-ni	itta
			athlete-NOM	10-time	gym-LOC	jump-PAST-COMP	student-TOP	friend-DAT	say-PAST
			“The student said to his/her friend that the athlete jumped ten times at the gym.”						

The sets of critical verbs were chosen based on previous studies investigating unstated compositional iterative meaning (e.g. Piñango et al. 1999; Paczynski et al. 2014). Specifically, the critical punctual verbs (e.g. *jump, cough, hop*) in PuncIter and GapIter denote instantaneous actions belonging to semelfactives in terms of lexical aspect (Comrie 1976; Smith 1994, 1997). The durative verbs (e.g. *run, sleep, swim*) in NoIter and DurIter denote atelic activities (i.e. action events without an inherent/natural endpoint). While unstated iteration is initially thought to be modulated by verb type (e.g. NoIter: *swam for an hour* vs. PuncIter: *jumped for an hour*), Deo and Piñango (2011) point out that the interval lengths denoted by the durative adverbial co-determine the ultimate reading (e.g. NoIter: *swam for an hour* vs. DurIter: *swam for 1 year*) as discussed in Introduction. We therefore take both factors into consideration in the stimuli, fully crossing the two for completeness. Note that, however, NoIter sentences engendered a one-time reading whereas the other three (PuncIter, DurIter, GapIter) engendered an unstated iterative reading. Since we focus on what neurocognitive mechanisms are involved in the computation of sentential unstated iterative meaning, we merged PuncIter, DurIter, and GapIter, as ITER in data analysis, to be compared with NoIter and sentences with transparent iterative readings (see below).

In addition, we introduced 20 sets of two transparent conditions with the frequentative adverbials *-kai* (*n*-times), which specified exactly how many times the denoted event occurred. The sentences of the Multiple condition directly stated the number of event iterativity (i.e. explicit iteration). This was in direct contrast with the three ITER conditions, in which the iterative meaning had to be computed from sentential context by comprehenders. On the other hand, the Once condition contained the frequentative adverbials *ikkai* 1-time `once.' It differed from the NoIter condition in that the one-time reading was explicitly specified in the Once condition (i.e. explicit once), and not so in the NoIter condition (i.e. unstated once).

By hypothesis, sentences of the ITER conditions all involved unstated iteration, and would show additional cortical activity than the Multiple condition that involved explicit iteration in regions supporting unstated meaning computation. Likewise, the NoIter condition (unstated once) might require more computational effort than the explicit Once condition for computing the one-time continuous reading of the sentences. Furthermore, the Multiple (explicit iteration) > Once (explicit once) contrast would inform numerosity processing via linguistic devices, i.e. frequentative adverbials.

All sentences were of the intransitive construction in the target clause, which contained the critical verb and the *kan*-adverbial, with an animate subject in both the target clause as well as the matrix clause. The four conditions with unstated meaning appeared in the following sentential frame: [[Subject NP-ga + Temporal *kan*-Adv. + Locative NP-de + Target Verb]_CP_-to + matrix Subject NP-wa + matrix Object NP-ni + matrix Verb] (*−ga*: Nominative case; *−de*: Locative case; −*ni*: Dative case; *−to*: Complementizer; −*wa*: topic marker). For the transparent Once and Multiple conditions, the temporal *kan-*adverbial (Seg.02) was replaced by a frequentative adverbial, *ikkai* (once) and n-*kai* (n-times), respectively. All sentences consisted of seven segments each as labeled in Table 1.

### Stimuli norming questionnaire

The 40 sets of the critical conditions (NoIter, PuncIter, DurIter, GapIter) were submitted to a norming questionnaire to ensure the sentences’ naturalness and interpretations. The questionnaire included 20 sets of two filler conditions, in which an inanimate subject (e.g. *the first chapter*) and an aspectual verb (e.g. *begin, finish*) were combined with an activity-denoting complement (Filler-1; e.g. *the editing*), or with an entity-denoting complement (Filler-2; e.g. *the novel*). Both fillers were deemed unacceptable in Japanese, thereby balancing the design for naturalness rating. The whole set of stimuli amounted to 200 sentences. The frequencies of the two verb types (punctual, durative) of the critical conditions were checked using mixed-effects model analyses implemented in the R statistical environment (R Core Team 2021) with the *lme4* package (Bates et al. 2015). Results showed no frequency difference between verb types in either the lemma form (*χ*^2^(1) = 1.70, *p* = 0.19) or the past-tense TA-form (used in the experiment) in Japanese (*χ*^2^(1) = 0.53, *p* = 0.46). Verb length did not differ either (*χ*^2^(1) = 0.66, *p* = 0.42).

We recruited 22 native speakers of Japanese (13 females, mean age = 20.55 y.o., range = 18 ~ 23 y.o.) who had no history of reading disability or language impairments by self-report. Informed consent was obtained from each participant prior to the questionnaire. The participants were asked to read each sentence, (i) rate its naturalness from a scale 1~5 (5 = *completely natural*, 1 = *completely unnatural*) and (ii) determine the iterativity of the event denoted by the critical verbal predicate among five options: (i) *once*, (ii) *more than twice*, (iii) *˃10 times*, (iv) *˃100 times*, (v) *the sentence does not make sense*.

**Fig. 1:**
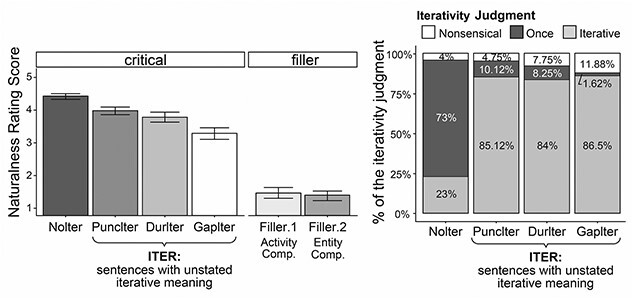
Left: Results of naturalness rating in the norming questionnaire (mean ± 1 standard errors). Right: Results of Iterativity judgments in the norming questionnaire (responses of “*more than twice*”, “*more than 10 times*”, and “*more than 100 times*” are grouped as the [iterative] response type).

Two participants were identified as outliers in data screening (behavioral responses falling outside of 2.5 standard deviations (SD) from the group mean) and their data were excluded from further analysis. Statistical analyses were performed using mixed-effects model analyses. Raw rating scores were transformed into *z*-scores to balance individual variation (Schütze and Sprouse 2014); data points showing |*z*| > 2.5 were excluded to minimize impact from extreme values. The models included Verb Type and adverbial Interval as the fixed factors; the random-factor structure incorporated an intercept for both subject and item-set, as well as the interaction of the two fixed factors over subject and item-set (i.e. *lmer*(Rating.z.score ~ VerbType^*^Interval + (1 + VerbType^*^Interval|Subject) + (1 + VerbType^*^Interval|ItemSet), data, REML = F). Effect significance was assessed via backward model comparisons, contrasting the fixed factor in question against a base model without it. Results showed that all critical conditions were rated within the natural range in average (>3.5), whereas the nonsensical fillers were rated below 2 (Fig. 1, left panel). The critical conditions showed a significant main effect of Interval (*χ*^2^(1) = 18.67, *p*< 0.001) and Verb Type (*χ*^2^(1) = 23.09, *p* < 0.001), but no interaction between the two (*χ*^2^(1) = 0.52, *p* = 0.47). Results of iterativity judgments revealed that the three ITER conditions of unstated iteration (PuncIter, DurIter, GapIter) received iterative readings mostly (>80%), while the NoIter condition received mostly the once reading (73%), shown in Fig. 1 (right panel).

For the ensuing fMRI experiment, we removed 8 sets of critical conditions that received relatively unexpected interpretations and replaced the two filler conditions with the two transparent baselines, Once and Multiple, for comparison (Table 1). The whole set of stimuli consisted of 32 sets of four critical conditions plus 20 sets of two transparent conditions, amounting to a total of 168 sentences.

### Participants

A total of 20 native speakers of Japanese were recruited (12 females; mean age = 23.63 y.o., SD = 3.44 y.o., range = 20~34; biographical data from one participant was missing), without reading disabilities by self-report; all had normal or corrected-to-normal vision. All participants were right-handed (*Mean* = 98.5; *SD* = 4.89; range = 80~100) by the Japanese version of the FLANDERS handedness questionnaire (Nicholls et al. 2013; Okubo et al. 2014). The study was approved by the ethics committee of the National Rehabilitation Center for Persons with Disabilities in Japan. Written informed consent was obtained from each participant prior to the experiment.

### Procedure

Sentences were visually presented segment-by-segment in black against a light-gray background projected onto an magnetic resonance (MR)-compatible liquid-crystal display (NordicNeuroLab, Bergen, Norway) with a resolution of 1,920 × 1,080 pixels at a refresh rate of 60 Hz. The screen stood near the head side of the MRI bore and participants viewed the screen through a mirror attached to the head coil. The participants were instructed to read each sentence silently and made an Iterativity judgment, for which they had to determine how many times the event denoted by the critical verbal predicate (e.g. *jump, run*) occurred among four options: (i) *once*, (ii) *2–10 times*, (iii) *11–100 times*, and (iv) *˃101 times*, in a timely manner. The participants responded using their index and middle fingers of the left and right hands. We counterbalanced two button assignments to the four answer options, namely left-middle (i), left-index (ii), right-index (iii), right-middle (iv), and the reverse order, i.e. right middle finger (i) ~ left middle finger (iv). A practice session was given to familiarize the participants with the procedure outside of the scanner prior to the real trials. Stimuli presentation and response recording of the behavioral data were implemented by the Presentation® software (Neurobehavioral Systems, Inc., Albany, CA, USA). The participants took AQ (Baron-Cohen et al. 2001) in its Japanese version (Wakabayashi et al. 2004) in a separate session on a distinct day of the imaging session.

### Paradigm

**Fig. 2:**
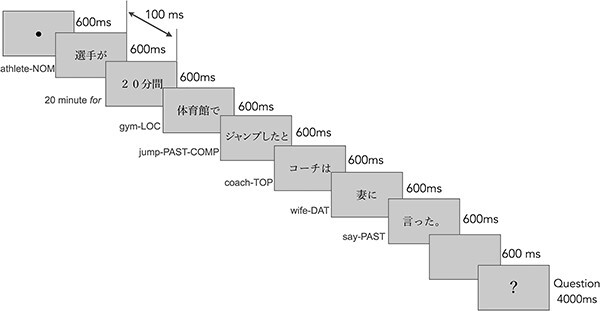
Trial procedure in the fMRI experiment. The translation texts at the left side of the frames were provided for illustration only and were not shown to the participants.

Each sentence began with an initial fixation point appearing at the center of the screen. Sentences were presented segment-by-segment, with seven segments per sentence; each segment remained on the screen for 600 ms, with 100 ms blank between consecutive segments (Fig. 2). Following the sentence, a question mark that prompted the iterativity judgment appeared on the screen for 4 s, with a 600 ms interval from the end of the sentence-final segment. Sentence onset asynchrony was jittered for 11, 12, 13, or 14 s (mean = 12.5 s) to reduce stimulus predictability for better estimation of hemodynamic response to the stimuli.

### Imaging acquisition

The imaging data were collected using a 3 T MRI scanner (MAGNETOM Skyra; Siemens, Erlangen, Germany).

*Anatomical measurements.* Axial slices covering the whole-brain were aligned to the anterior commissure–posterior commissure (AC–PC) plane or to the slightly tilted planes than AC–PC plane. T1-weighted high-resolution structural images were acquired using Magnetization Prepared Rapid Gradient Echo sequence (Repetition Time (TR) = 2,300 ms, Echo Time (TE)  = 2.98, inversion time = 900 ms, flip angle = 9°, field of view = 256 × 256 mm^2^, matrix 256 × 256, sagittal 224 slices, 1 mm isotropic resolution).

*Functional measurements*. We obtained fMRI data using a multiband-accelerated echo-planner imaging sequence (Moeller et al. 2010) in one session consisting of 2,105 volumes (⁓40 min, TR = 1,000 ms, TE = 30 ms, flip angle = 68°, field of view = 192 × 192 mm^2^, matrix 64 × 64, 30 axial slices, slice thickness = 3 mm with a 1 mm gap, and multiband acceleration factor = 2).

## Data analysis

Prior to statistical analysis of the imaging data, we screened the behavioral responses of the participants. Two participants were excluded from further analysis, one for response rate < 75% and the other for being identified as an outlier (whose responses to the Iterativity judgments were undifferentiable across the critical conditions, falling outside of three SD from the group mean in two critical conditions, and showed negative inter-subject correlations with all the other participants). Thus, a total of eighteen subjects’ data were submitted to the following statistical analysis.

### Whole-brain imaging data analysis

The imaging data were preprocessed by the Statistical Parametric Mapping 12 software package (available at http://www.fil.ion.ucl.ac.uk/spm/) in the MATLAB environment (version 2021a). The functional images were realigned to the first image and corrected for slice timing using the middle slice as the reference. The functional images were then co-registered to individuals’ structural images and spatially normalized to the East Asian brain template for group analyses. All images were registered to the Montreal Neurological Institute (MNI) space. The resulting images were resampled into a 3 × 3 × 3 mm^3^ voxels with the 7th-degree B-spline interpolation and spatially smoothed with a 6 mm full width half maximum Gaussian kernel to account for individual variation in activation location.

Statistical analysis of the fMRI data was performed using general linear models, with regressors for each condition, one regressor for trials with no response, and six regressors for head motion parameters. The group-level analysis was performed by the Statistical nonParametric Mapping (SnPM) 13 software package (toolbox available at http://www.nisox.org/Software/SnPM13/). We contrasted the three iterative conditions (PuncIter, DurIter, GapIter) grouped as ITER (unstated iteration), with the two baseline conditions, Multiple (explicit iteration) and NoIter (unstated once), respectively. These revealed the neural correlates of the computation of unstated combinatorial meaning. We also contrasted the NoIter condition (unstated once) with the Once condition (explicit once), which would reflect unstated meaning processing, to investigate if it shared common regions with the “ITER > Multiple” contrast. In addition, we compared the Multiple condition with the Once condition for the computation of explicit iteration, which also allowed a comparison with unstated iteration. Variance smoothing was set to [8 8 8]. The number of permutations was set to 10,000. Results are reported with thresholding at cluster-level family-wise error corrected at *P*_FWE-corr_ < 0.05.

### Assessment of the correlations between individual AQ scores and ROI activity

To investigate if individual socio-cognitive propensity indexed by AQ co-varied with the activation of the brain regions engaging in processing unstated meaning, we computed Pearson correlation coefficients between the activation level (beta-values) of the Regions-Of-Interest (ROIs) and individuals AQ scores. The center of spherical ROIs (*r* = 6 mm) were chosen from the maxima of activated clusters identified in the whole-brain analysis across the designated contrasts (Table 2) using Marsbar 0.45 (Brett et al. 2002). Regions showing correlation coefficients |*r*| ≥ 0.4 between AQ and regional activation are reported and discussed, given that *r* with an absolute value ≥0.4 is considered a medium to large effect (Field et al. 2012:209; Hinkle et al. 2003).

**Table 2:** Imaging results of the whole-brain analysis.

Contrast	Region	MNI coordinate [x, y, z]	Volume (k_E_)	*T*-values
**ITER > Multiple** Unstated meaning =Unstated iteration – Explicit iteration	L.frontal sup.medial region/ L.SMA (BA 8)	[−3, 23, 41]	1420	7.73
	L.precuneus/retrosplenium	[−3, −52, 14]	379	7.44
	L.Vermis	[−3, −55, −34]	75	4.40
	R.Vermis	[3, −79, −19]	298	5.81
**ITER > NoIter** Unstated Iteration = Unstated iteration – Unstated once	R.SFS (BA 6)	[27, 14, 56]	142	6.67
	R.AG (BA 39)	[45, −64, 32]	102	5.87
	R.IPS (BA 7)	[39, −37, 38]	113	4.59
**Multiple > Once** Explicit iteration = Explicit iteration – Explicit once	L.IPS	[−27, −64, 38]	669	6.86
	R.IPS/AG (BA 39)	[36, −61, 50]	461	6.80
	L.PT	[−42, 44, −4]	92	5.17
	L.SFS	[−24, 14, 47]	89	4.44
**NoIter > Once** Unstated meaning = Unstated once – Explicit once	L.SMA	[−3, 20, 44]	1297	7.64
	L.IPS/L.SMG (BA 40)	[−48, −37, 47]	346	6.37
	R.Cerebellum	[36, −64, −40]	91	5.25
**Single condition contrasts between the NoIter, PuncIter, DurIter, and GapIter conditions**				
**PuncIter > NoIter** Unstated Iteration	R.SFS (BA6)	[27, 14, 56]	180	7.03
	R.PT	[48, 35, 5]	191	5.37
	L.PT	[−45, 38, 8]	169	5.29
	R.AG (BA 39)	[45, −67, 32]	211	5.11
**PuncIter > DurIter**	L.SMG	[−63, −34, 26]	137	5.91
	L.PT	[−48, 38, 8]	153	5.18
	R.PO	[51, 17, −7]	66	4.49
	R.SMG	[54, −40, 41]	156	4.37
	R.MTG	[57, −16, −10]	86	4.34
**PuncIter > GapIter**	L.PT	[−45, 38, 8]	154	6.15
**GapIter > NoIter**	R.POS (BA23)	[18, −55, 20]	245	4.62
**GapIter > DurIter**	R.pSTS (BA22)	[48, −43, 11]	45	4.19

*Note:* Thresholded at *p* < 0.05 family-wise error corrected for multiple comparisons at the cluster level. L, left; R, right; PT, pars triangularis; PO, pars opercularis; IFJ, inferior frontal junction area; AG, angular gyrus; SFS, superior frontal sulcus; IPS, intraparietal sulcus; SMA, supplementary motor area; SMG, supramarginal gyrus; DLPFC, dorsal-lateral prefrontal cortex. POS, parieto-occipital sulcus.

In particular, we targeted the ITER vs. Multiple contrast (in regional beta-values) that reflected the computation of unstated meaning, examining if the ROIs would show differential activation varying with individuals’ AQ scores. Since previous studies reported that autistic individuals exhibited decreased activation in the left inferior frontal area during sentence processing (e.g. Just et al. 2004; Mody and Belliveau 2013; Williams et al. 2013), we expected a negative correlation between AQ and the regional activation in the left frontal region during the computation of unstated meaning. Furthermore, we compared this variability in unstated iteration with the Multiple vs. Once contrast (in regional beta-values) that reflected the processing of explicit iteration. Distinct correlation patterns were expected in these two contrasts due to the nature of combinatorial processing, namely unstated vs. explicit meaning computation.

## Results

### Behavioral results: Iterativity judgments

**Fig. 3:**
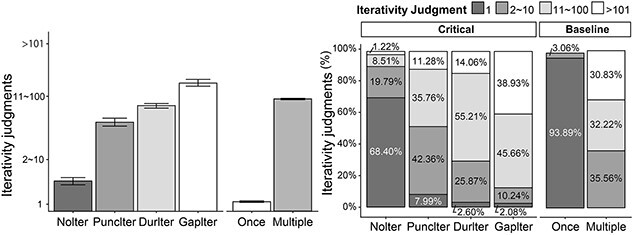
Iterativity judgments in the fMRI scanning session (*n* = 18). The participants determined how many times the event denoted by the verbal predicate occurred in the sentential context after reading, among four options: (i) *once*, (ii) *2–10 times*, (iii) *11–100 times*, or (iv) *>101 times*. Left: Averaged responses using the options (i)–(iv) across participants. Right: The proportions of each response option chosen per condition.

Results showed a graded pattern of iterativity reading across the four conditions with *kan-*adverbials. The NoIter condition elicited mostly the “once” response, whereas the three ITER conditions received the iterative responses for the majority of the trials (Fig. 3). The two transparent conditions with frequentative *kai*-adverbials, Once and Multiple, induced mostly the once and iterative readings respectively as expected.

**Fig. 4:**
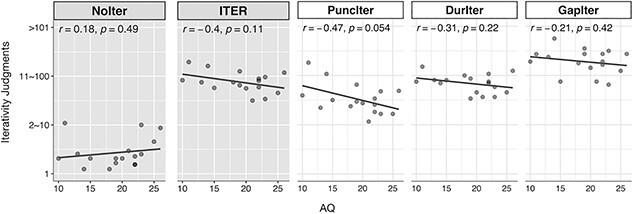
Correlations between individual AQ scores and iterativity judgments in the fMRI session (*n* = 18). The PuncIter, DurIter and GapIter conditions were grouped as ITER. Pearson correlation coefficients were computed between each participant’s AQ scores and iterativity judgments (*once*, *2–10 times*, *11–100 times*, *>100 times*) per condition.

In addition, we computed the Pearson correlation coefficients between individual AQ scores and iterativity judgments (using the response options (i)–(iv), representing “*once*,” “*2–10 times*,” “*11–100 times*,” and “*more than 101 times*,” respectively) for the three ITER conditions involving unstated iteration. We performed this behavioral analysis using the *stat_corr*() function of the ggpubr package in the R statistical environment. We took a correlation coefficient |*r*| ≥ 0.4 as indicating a moderate to strong effect (Field et al. 2012:209; Hinkle et al. 2003). Results showed that AQ scores negatively correlated with iterativity judgments in the three ITER conditions (*r* = −0.4), as shown in Fig. 4. The effect was more prominent in the PuncIter condition (*r* = −0.47) while weaker at the other two conditions (DurIter: *r* = −0.31; GapIter: *r* = −0.21). The pattern suggests that the higher the AQ, the less a comprehender estimated the number of event iterativity when the iterative meaning was not explicitly stated.

### Imaging results of the whole-brain analyses

**Fig. 5:**
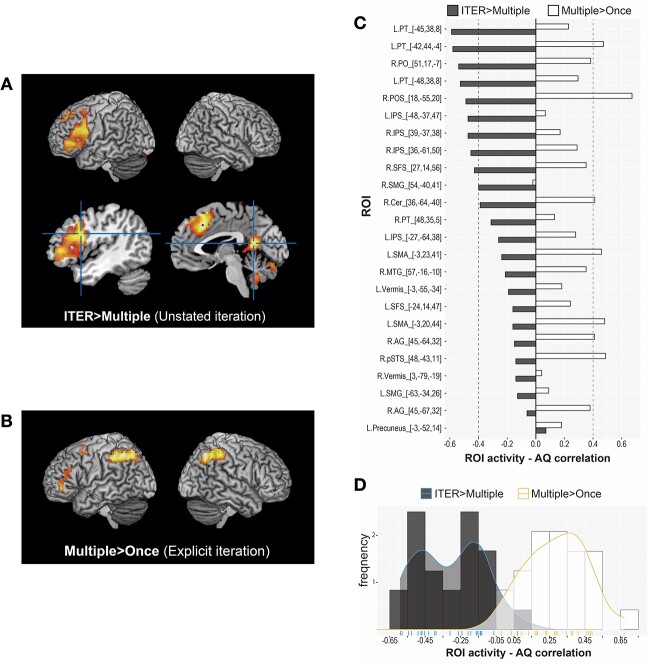
Imaging results of the whole-brain activation in the (A) “ITER > Multiple” and (B) “Multiple > Once” contrasts. (C) Correlations between the individual brain activation (beta-value) and AQ in the “ITER > Multiple” and “Multiple > Once” contrasts across all ROIs. The ROIs were chosen by the maxima of the activated regions in the contrasts identified in the whole-brain analysis (Table 2). (D) The distributions of the ROI-AQ correlations in the ROIs reported in (C), for the “ITER > Multiple” (black) and “Multiple > Once” (white) contrasts.

The “ITER > Multiple*”* contrast, reflecting unstated meaning processing, preferentially recruited the left medial frontal areas at the vicinity of the supplementary motor area (SMA) and left inferior frontal junction (L.IFJ), bilateral precuneus, and bilateral vermis (Fig. 5A). The “NoIter > Once*”* contrast reflecting unstated meaning processing showed similar recruitments of the L.SMA and L.IFJ, left intraparietal sulcus (L.IPS)/supramarginal gyrus (L.SMG) and the right cerebellum. The “ITER > NoIter” contrast, reflecting the computation of unstated iteration, showed greater activation in the right superior frontal sulcus (R.SFS), right angular gyrus (R.AG), and the right intraparietal sulcus (R.IPS). In comparison, the “Multiple > Once” contrast (Fig. 5B), which reflected the processing of explicit iterativity, preferentially elicited the bilateral IPS including the R.AG, L.PT, and the left superior frontal sulcus (L.SFS). At the level of single-condition contrast, the L.PT was prominent for computing the unstated iteration especially in the PuncIter condition. The GapIter condition induced greater activation in the right parieto-occipital sulcus (R.POS) and the right posterior superior temporal sulcus (R.pSTS) than the NoIter and the DurIter condition, respectively. Results of the activation maxima are reported in Table 2.

### Imaging results: Brain regions sensitive to individual AQ variability

The contrasts of “ITER > Multiple” and “Multiple > Once” in regional beta-values showed opposite directions in the ROI-AQ correlations (Fig. 5C), with negative correlations in “ITER > Multiple” for unstated meaning yet positive correlations in “Multiple > Once” for explicit iteration. The effect was especially prominent in the L.PT and the IPS-POS regions (|*r*| ≥ 0.4). These suggested that individual variability in AQ, indexing socio-cognitive propensity, varied with the processing of unstated combinatorial meaning and that of explicit iteration in opposing manners (Fig. 5D).

## Discussion

In this study, we examined the neurocognitive mechanisms of processing unstated meaning at the compositional level in real-time. The comprehension of combinatorial unstated meaning is considered to include two subcomponents: a context-dependent meaning search in sentential-discourse context and the enrichment of detailed information, i.e. the calculation of event numerosity in the current case. The results suggested that the meaning search in sentential context was subserved by the left inferior frontal regions, while enriching the detailed event iterativity recruited bilateral medial and lateral parietal regions including the precuneus/retrosplenial and IPS. Moreover, we investigated the impact of individual socio-cognitive propensity on such meaning processing by probing the correlations between the regional brain activations and individual AQ. In the L.PT, we found negative correlations with AQ for computing unstated meaning yet positive correlations for explicit meaning, demonstrating an influence of individual socio-cognitive propensity in the comprehension of combinatorial meaning. We suggest accounting for such individual variability in the implicit vs. explicit meaning computation by the DPA of information processing (e.g. Brosnan et al. 2016; Evans 2003; Kahneman 2011; Rozenkrantz et al. 2021).

### The computation of combinatorial unstated meaning: The left inferior frontal regions and the parietal cortex

The processing of unstated meaning manifested by the “ITER > Multiple” contrast (unstated iteration > explicit iteration) preferentially recruited a fronto-parietal network, including the L.superior medial frontal cortex, L.IFJ, bilateral precunei, and the retrosplenial cortex. A similar left-lateralized fronto-parietal activity was found in the “NoIter > Once” contrast (unstated once > explicit once), in the L.IFJ and L.IPS/SMG. Since the comprehension of combinatorial unstated meaning involves a meaning search in the sentential context, we suggest that it relies on the (relatively left-lateralized) fronto-parietal network. This fronto-parietal network has been shown in pragmatic processing involving nontransparent meaning, such as indirect speech (van Ackeren et al. 2012). Accordingly, we reason that the current results likely manifest a network for meaning contextualization, i.e. meaning search in the sentential-discourse context for specifying the unstated information.

The L.PT in the fronto-parietal network crucially responds to the conceptual-contextual search for unstated iteration. When the meaning search is deeper, especially when the target (intended) meaning is not explicitly given by linguistic input, the processing load is higher and the L.PT activation increases (Badre et al. 2005; Rodd et al. 2010; Husband et al. 2011; Piñango et al. 2017; Lai et al. 2020). The L.PT has been argued to contribute to semantic processing while the left pars opercularis subserves syntactic structure building (Friederici et al. 2000; Makuuchi et al. 2009, 2012; Glaser et al. 2013; Schell et al. 2017; Zaccarella et al. 2017). The L.PT also shows higher activation in the search and evaluation of sentential meaning in context (Poldrack et al. 1999; Vigneau et al. 2006; Zhu et al. 2009, 2013; Rapp et al. 2012; Lai et al. 2017). In fact, the L.IFG (including L.PT) emerges in the majority of studies on figurative language, such as metaphor (e.g. Eviatar and Just 2006; Prat et al. 2012), metonymy (Rapp et al. 2004, 2011; Piñango et al. 2017), and irony (Wang et al. 2006; Regel et al. 2011; Spotorno et al. 2012), as compared to the literal counterparts. These findings are in line with the view that the L.PT and L.IFJ subserve meaning contextualization in the current study, such that a deeper contextual search engages stronger activation in the frontal regions.

On the other hand, the parietal regions may subserve “the context-based activation of lexical and world knowledge” (Baggio 2018:158), indicated by several studies (Bookheimer 2002; Friederici et al. 2003; Rodd et al. 2005). We speculate that the precuneus and the retrosplenial cortex (Fig. 5A) are parts of the network for scene construction (Hassabis and Maguire 2007; Ma et al. in prep; Sulpizio et al. 2016; Vann et al. 2009), likely supporting episodic memory (Wagner et al. 2005; Binder et al. 2009; Binder and Desai 2011). To obtain the unstated event iteration, comprehenders may consult their own experience to determine how many times the denoted event (e.g. *jump*) would take place, given the time-interval specified by the durative adverbial (e.g. *5 minutes*). Mental reconstruction of the event scene or episodic memory retrieval could support these cognitive processes. The AG (shown in the “ITER > NoIter” contrast), which has been suggested as a hub for event knowledge by Binder and Desai (2011), likely engaged in the event conceptualization as well (cf. Seghier 2013). They also indicate that the posterior cingulate gyrus and the adjacent precuneus “may function as an interface between the semantic network and the hippocampal memory system, helping to encode meaningful events into episodic memory” (*p.*531).

We found greater IPS activation in the “Multiple > Once” contrast (reflecting the computation of explicit iteration, bilateral IPS) and “ITER > NoIter” (reflecting the computation of unstated iteration, right-lateralized IPS). These results are in line with previous studies which identify the IPS as a core region supporting number or quantity processing (Carreiras et al. 2010; Dehaene et al. 2003; Dormal et al. 2008; Holloway et al. 2010; Piazza and Izard 2009). In the current study, the increased IPS activity for iterative interpretations was found for not only explicit iteration (“Multiple > Once”) but also unstated iteration (“ITER > NoIter”). These paralleled patterns suggest that the IPS subserves computation of iterativity that emerges combinatorially. Meanwhile, the activations in IPS and IPL (including SMG) have showed sensitivity to the processing of numbers (Amalric and Dehaene 2018; Dehaene et al. 2003), duration-tuned representations (Hayashi et al. 2015) and temporal expectations (Bueti et al. 2010). Incorporating these findings, we suggest that the IPS subserves the computation of event iterativity that enriches the unstated meaning. The numerosity processing in the IPS seems unlikely to be bound specifically to explicitly given numbers, yet possible to be triggered by numerosity that frequentative adverbials indicate in a combinatorial context.

**Fig. 6:**
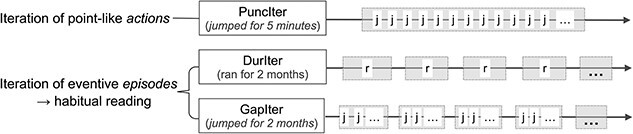
Illustration of the iterative meaning in the PuncIter, DurIter, and GapIter conditions.

While the frontal–parietal activation emerged in the three iterative conditions grouped as the ITER (being compared with the Multiple or NoIter condition), at the level of single-condition contrast we observed the highest L.PT activation for the PuncIter condition (e.g. *jumped for 5 minutes*), compared with both the DurIter (e.g. *ran for 2 months*) and the GapIter conditions (e.g. *jumped for 2 months*). This suggests that the iterative meaning in the PuncIter condition may differ qualitatively from the DurIter and GapIter conditions, though all three involve unstated iterative meaning. Examining the semantic representation more closely, we note that the PuncIter sentences involve iterative actions occurring in one single episode, whereas the DurIter and the GapIter counterparts involve iterative actions occurring across multiple episodes (Fig. 6; also see Deo and Piñango 2011). It could be that the PuncIter condition indicates the repetition of point-like *events*, while the DurIter and GapIter conditions involve the repetition of eventive *episodes*, which further implicate a habitual interpretation (e.g. *Sam used to run for 2 months.*); hence the differences.

We also found that the GapIter condition induced greater activation in the R.POS (parieto-occipital sulcus) and the R.pSTS (posterior superior temporal sulcus) than the NoIter and the DurIter conditions, respectively. The POS together with the neighboring retrosplenial cortex have been indicated to support imagined events pertaining to personal autobiographical memory (e.g. Vann et al. 2009; Epstein et al. 2017) and scene construction (e.g. Silson et al. 2019). We suggest that the GapIter condition engendered both iteration of point-like actions and iteration of episodes spanning across a longer interval (Fig. 6). It is therefore not surprising that the GapIter recruited areas supporting imagined scene construction to a greater extent than the DurIter (iteration of episodes without the iteration of point-like actions) and the NoIter (no iteration) conditions.

Besides, it is possible that the RH activation (the R.SFS, R.AG, and R.IPS) for unstated iterative meaning (“ITER > NoIter”) reflected greater difficulty in comprehension. The RH homolog of the language network has been reported to activate during the processing of inference (Mason et al. 2008), irony (Wang et al. 2006), and speaker-incongruent sentences (Tesink et al. 2011). Fonseca et al. (2009) and Federmeier and Kutas (1999) indicate that the RH co-activation contributes to discursive, pragmatic-inferential processing. For instance, metaphorical expressions have often been reported to recruit the right frontal regions (e.g. Ahrens et al. 2007; Schmidt and Seger 2009) as well as the right posterior temporal regions (Eviatar and Just 2006; Mashal et al. 2009), especially when the metaphors are unfamiliar (Lai et al. 2015). With context, the processing of sentential metaphors in two-sentence texts also recruits the R.IFG and right anterior temporal regions (Diaz and Hogstrom 2001). Similarly, studies on metonymy reported activation in the R.IFG in addition to the L.IFG (Rapp et al. 2011: BA45/47). Others have proposed that the RH activation in language processing increases with integration demands while the recruitment is not limited to figurative language (Diaz and Eppes 2018). In light of these studies, the RH activation could plausibly result from a costlier meaning-mining process for enriching the unstated iteration.

### Autistic traits (AQ) modulate brain activation for the processing of unstated meaning in the L.PT and IPS/POS

In addition to the general pattern of unstated meaning processing, the activation level of the fronto-parietal network negatively correlated with individual socio-cognitive propensity indexed by AQ (Fig. 5C and D). Following previous studies (Zhang et al. 2022; Lai and Lai 2023), we hold that individuals with higher AQ scores (indexing lower social skills) are weaker at meaning contextualization and less prone to obtain unstated meaning in the iterativity judgments (Fig. 4); this was accompanied by lower activations in the L.PT and IPS-POS revealed by the “ITER > Multiple” contrast (Fig. 5C). That is, less efficient meaning contextualization may lead to lower capacity of mining numeral-temporal information during the computation of unstated iterative meaning. Here we address the issue by examining the impact of individual context sensitivity indexed by AQ in real-time language comprehension.

Above we have suggested that the process of enriching unstated meaning (“ITER > Multiple”) is in part supported by the left frontal regions including the L.PT at the group-level analysis; the results of ROI analyses further revealed that higher AQ scores were associated with lower L.PT activation. Crucially, the AQ scores and L.PT activity showed a positive correlation in the “Multiple > Once” contrast that reflected the computation of explicit iteration. These combined suggest that the negative L.PT–AQ correlation reflects the computation of unstated meaning *per se*, revealing a less efficient processing in higher-AQ individuals, rather than the iterativity/numerosity computation (which engages the IPS). This view is consistent with the previous findings that individuals with ASD exhibited weaker L.IFG activation during nonliteral language comprehension (e.g. Mason et al. 2008; Groen et al. 2010), such as metaphor (see Kalandadze et al. 2019 for a review) and irony (Wang et al. 2006; Williams et al. 2013). Our findings suggest a lower degree of contextual integration in higher-AQ TDs as well, underpinned by a weaker L.PT function during combinatorial language processing.

The DPA may provide an insightful account for the correlation between individual AQ scores and the L.PT-IPS/POS activation. On the DPA, individuals’ cognitive styles differ in the relative reliance on deliberation vs. intuition during information processing (Evans 2003; Evans 2010; Kahneman 2011; Brosnan et al. 2016; Rozenkrantz et al. 2021). People with higher autistic-like traits (i.e. lower socio-cognitive propensity) are more prone to deliberative reasoning and rule-based computation using local, explicit cues (cf. Ashwin and Brosnan 2019). This is accompanied by less sensitivity to contextual information in the larger sentential-discourse environment. Accordingly, obtaining the combinatorial unstated meaning is more effortful for higher-AQ individuals due to their lower context sensitivity, and this is correlated with lower activation of the fronto-parietal network. For higher-AQ individuals, lower L.PT activity may signal less intuitive and less integrative manners during information processing, corresponding to lower context sensitivity. Connectedly, the lower IPS activation suggests a weaker degree of information-mining processes for detailed iterativity and duration-tuned representation in sentential context when the target meaning is unstated. It could be that a weaker tendency of meaning contextualization hampers further computation processes (probably because higher-AQ individuals do not proceed to the further step). On the DPA, the greater reliance on the rule-based computation by explicit input comes with diminished higher-order computation (Baron-Cohen et al. 2009; Greenberg et al. 2018; Mottron et al. 2006). This could hinder the inference of unstated iterativity, as shown by the negative correlation of AQ and iterativity judgments (Fig. 4).

The DPA provides an integrative interpretation of the opposing correlation patterns, namely the negative correlation between AQ scores and the frontal–parietal activation for unstated meaning processing (“ITER > Multiple”) and the positive correlation between AQ and this network for explicit meaning processing (“Multiple > Once”) (Fig. 5C and D). The positive correlation suggests that the L.PT and IPS/POS react more to explicit numerosity given by clear linguistic cues (n-*kai* “n-times” in the Multiple condition) in higher-AQ individuals. In other words, higher-AQ individuals may be more willing to compute explicitly specified numerosity of events, but less motivated to mine the meaning when it is unstated and has to be computed by contextual search. The positive vs. negative correlations with AQ scores in this frontal–parietal network for explicit/stated vs. implicit/unstated meaning respectively correspond to the preference for rule-based deliberative computation vs. heuristic processing of the DPA.

The DPA seems highly compatible with the Good-Enough approach that proposes two independent routes in language processing (Ferreira et al. 2002; Karimi and Ferreira 2016). Karimi and Ferreira (2016) note that “linguistic representations are formed through a complex interplay between simple heuristics and deep syntactic algorithms (*p.*1013).” When processing sentences, comprehenders seek to form a meaningful, coherent representation to achieve cognitive equilibrium as early as possible. The algorithmic route works in a bottom-up fashion, using given input and well-defined linguistic rules. The heuristic route relies on top-down information from semantic memory. The two routes are independent to each other yet work interactively to accomplish the language comprehension task. Similar dual-stream models of semantic processing have been proposed by Kuperberg (2007) and Baggio (2018:24) as well. In both studies, language processing consists of a top-down heuristic/synthesis mechanism that pre-activates semantic features for meaning computation and a bottom-up algorithmic/analytic mechanism that computes meaning based on syntactic rule. These dual-route accounts to language processing can be regarded as an instantiation of DPA to information processing; all involve a faster heuristic/intuitive route by top-down predictive inferences and a slower algorithmic/deliberative route that counts on explicitly given input to deal with information (be it linguistic or non-linguistic). Comprehenders implement the two routes with differential weightings, characterizable by socio-cognitive propensity as indexed by AQ, leading to differential interpretive behaviors and cortical recruitments.

Taken together, the findings shed light on the neurocognitive systems of combinatorial semantic processing of unstated meaning. The full comprehension of unstated iterative reading requires sufficient sensitivity to sentential context (e.g. the verbal predicate and the interval denoted by the adverbial) and an integration with world knowledge (e.g. how many times would an event occur within a given interval). Individuals vary in context sensitivity and the strategies they use to achieve the appropriate interpretation, as we found that the processing profiles of unstated meaning vary with individual AQ scores. Such individual variability correlates with the activation of the fronto-parietal network including the L.PT and IPS/POS. We suggest that the individual differences reflect variability in cognitive styles during combinatorial semantic processing: individuals with higher AQ scores are weaker at meaning contextualization, showing lower activation in the left frontal regions. This could in turn hinder further meaning-mining process for computing unstated event iterativity, associated with impoverished activations in the IPS/POS. Our study suggests how this individual cognitive tendency is manifested in language comprehension and reveals the neural underpinnings of combinatorial meaning processing.

## Author contributions

Yao-Ying Lai (Conceptualization, Data curation, Formal analysis, Funding acquisition, Investigation, Methodology, Project administration, Visualization, Writing—original draft, Writing—review & editing), Hiromu Sakai (Conceptualization, Funding acquisition, Methodology, Resources, Writing—review & editing), and Michiru Makuuchi (Conceptualization, Data curation, Formal analysis, Funding acquisition, Investigation, Methodology, Project administration, Resources, Visualization, Writing—review & editing)

## Funding

This work was supported by the *Ministry of Science and Technology (MOST)* of Taiwan (currently named National Science and Technology Council, NSTC), grant number MOST 111-2410-H-004-097 to Yao-Ying Lai, Japanese Society for the Promotion of Science *(JSPS)* KAKENHI grant number JP17H06380 to Michiru Makuuchi. Hiromu Sakai’s effort in this project was supported by JSPS Grant-in-Aid for Challenging Exploratory Research grant number 18K18515 and Grant-in-Aid for Scientific Research(S) #23H05493.

*Conflict of interest statement:* The authors declare no conflicts of interest for this study.

## Data availability

The data are available upon request.
